# Progress of molecular targeted therapy for head and neck cancer in clinical aspects

**DOI:** 10.1186/s43556-021-00032-5

**Published:** 2021-05-30

**Authors:** Kenji Nakano

**Affiliations:** Department of Medical Oncology, Cancer Institute Hospital, Japanese Foundation for Cancer Research, Ariake, Tokyo, 135-8550 Japan

**Keywords:** Head and neck cancer, EGFR, PD-1, BNCT, Near-infrared photoimmunotherapy

## Abstract

Since the body’s head and neck area affects many functions such as breathing, swallowing, and speaking, systemic treatments to head and neck cancer patients are important not only for survival but also for preserving functions and quality of life. With the progress that has been made in molecular targeted therapy, anti-EGFR antibody (cetuximab) and immune checkpoint inhibitors (nivolumab, pembrolizumab) have provided survival benefits to head and neck cancer patients and are approved for clinical practice. Clinical trials incorporating these new drugs for patients with locally advanced head/neck cancers are underway. However, the existing clinical evidence regarding molecular targeted drugs for head and neck cancers is based mostly on clinical trials allocated to squamous cell carcinoma patients. New targeted therapies for non-squamous cell carcinoma patients were recently reported, e.g., tyrosine kinase inhibitors for the treatment of thyroid cancers and HER2-targeted therapy for salivary gland cancers. With the goal of improving local control, molecular targeted treatment strategies as salvage local therapy are being investigated, including boron neutron capture therapy (BNCT) and near-infrared photoimmunotherapy (NIR-PIT). Herein the history and landscape of molecular targeted therapy for head and neck cancers are summarized and reviewed.

## Introduction

**Fig. 1:**
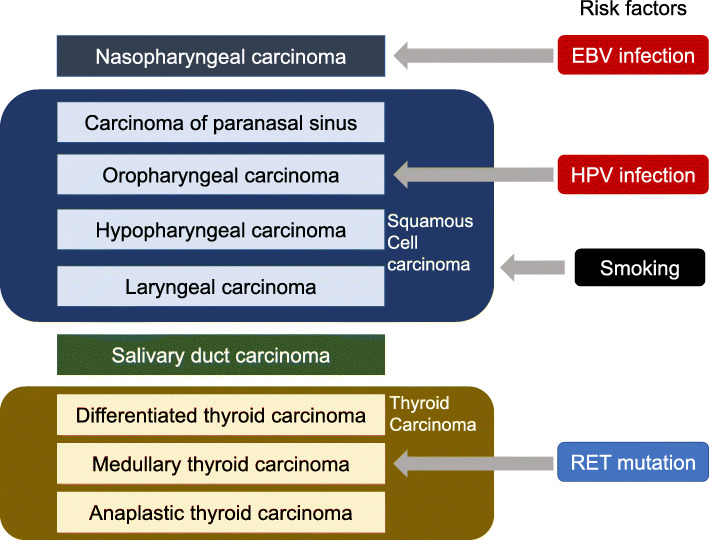
The Variety and risk factors of head and neck cancers. Smoking is the most facous risk factor for Squamous cell carcinoma of the head and neck (SCCHN), and viral infections such as human papilloma virus (HPV) and Epstein-Barr virus (EBV) are known to be risk factors for specific head and neck cancers. In thyroid cancers, RET mutation is known to have a close relationship with medullary thyroid carcinoma

Head and neck cancers, which originate in the oropharynx, hypopharynx, larynx and other areas between the supraclavicular areas and skull base, account for approx. 3% of all malignant diseases, and squamous cell carcinoma (SCC) is the most common histology [1]. Smoking has long been known as a risk factor for SCC of the head and neck (SCCHN), and viral infections have been recognized as a risk factor of head and neck cancers; e.g., the relationships between human papilloma virus (HPV) and oropharyngeal cancer and between Epstein-Barr virus (EBV) and nasopharyngeal cancer [2, 3]. The variety and risk factors of head and neck cancers are summarized in Fig. 1.

Approximately 60% of SCCHN patients are diagnosed at the locally advanced stage with complaints of neck swelling, hoarseness, difficulty in swallowing and more [1]. Curative surgical resection has been the standard treatment for SCCHN (with the exception of nasopharyngeal cancer), but this surgery can result in dysfunctions of swallowing, speaking, and even vision and hearing, and it can also cause cosmetic problems. There has been a great need for non-surgical treatment strategies that preserve the organ functions of the head and neck, and systemic chemotherapy and radiotherapy have thus been investigated as organ-preserving treatments.

**Fig. 2:**
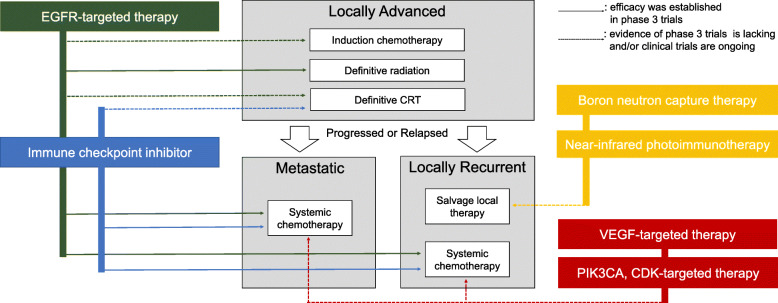
Landscape of non-surgical treatment strategy for SCCHN. In locally advanced patients, multimodal therapy including radiation and systemic chemotherapy are the standard regimen. Clinical evidence of molecular targeted drugs to these patients is limited yet. In progressed and/or relapsed setting, on the other hand, systemic chemotherapy is the standard and there are newly approved or investigating molecular targeted drugs. Some recurrent patients have locally recurrent diseases, to whom new local treatments have been investigated

Even when the primary lesion has been successfully treated, the cancers of some patients recur locally or metastatically, potentially resulting in death due to the cancer. The most common recurrence sites and events have been local or distant relapse, and lung, bone or liver metastases [4]. For relapsed/metastatic SCCHN patients, systemic chemotherapy is administered with the goal of survival improvement. The progress in molecular targeted therapy and cancer immunotherapy have extended the survival of SCCHN patients, particularly in the recurrent/metastatic setting. This review summarizes the development of molecular targeted therapy for head and neck cancer, focusing on the mechanisms underlying the treatment action and clinical role of each drug based on clinical evidence. The landscape of treatment strategy for head and neck cancer which would be discussed in the review, focusing SCCHN, was summarized in Fig. 2.

## Era of pre-molecular targeted therapy: cytotoxic chemotherapy

Before the emergence of molecular targeted therapy, systemic therapies for SCCHN had been conducted using cytotoxic antitumor drugs in platinum-based regimens, and today platinum-based regimens remains as the mainstream of systemic standard therapy for SCCHN [4, 5].

For patients with locally advanced SCCHN, systemic chemotherapy as an organ-preserving treatment strategy has replaced surgical resection and improved patients’ survival; for example, the combination of cisplatin and 5-fluorouracil (5-FU) for locally advanced laryngeal patients provided survival that was non-inferior to that afforded by surgery in a randomized clinical trial conducted in the 1990s [6]. The incorporation of systemic chemotherapy to enhance the synergy with radiation therapy is also being evaluated, and concurrent chemoradiotherapy (CRT) with high-dose cisplatin has been regarded as the standard non-surgical treatment for locally advanced SCCHN [7]. The clinical significance of CRT was also established for adjuvant settings after surgical treatment, especially for patients with positive surgical margins or extracapsular extension [8, 9]. The appropriate indications for and optimal treatment dose of cisplatin have been under investigation and remain a matter of controversy [10]. The addition of more cytotoxic therapy with induction chemotherapy and/or CRT has been tried, and three-drug combination chemotherapy with platinum, 5-FU, and taxane prolonged the patients’ overall survival (OS) compared to two-drug chemotherapy (platinum and 5-FU) in a locally advanced setting [11, 12]. However, the superiority of induction chemotherapy to CRT with cisplatin has not been established [13, 14].

For patients with recurrent/metastatic SCCHN, palliative systemic chemotherapy is the standard. Platinum is regarded as the key cytotoxic drug in this setting (as it is in locally advanced settings), and several cytotoxic agents with different mechanisms of action such as methotrexate and bleomycin as well as 5-FU and taxanes have been considered as candidates for palliative chemotherapy regimens [5]. Intensive combination chemotherapies were also considered for recurrent/metastatic settings [15–17], but although they showed high response rates these strategies provided limited clinical benefits in terms of survival. The performance status (PS) of SCCHN patients in a recurrent/metastatic setting is generally worse than that of patients in a local recurrent setting, and thus intensive cytotoxic therapy might result in the deterioration of patients’ quality of life (QOL) in exchange for a temporary response. Sequential monotherapy applied to recurrent/metastatic SCCHN, especially as a second or later line of treatment, has been preferred considering patients’ PS [5]. In general, the OS of recurrent/metastatic SCCHN patients has been approx. 6–9 months.

## EGFR-targeted therapy

The era of molecular targeted therapy began at the start of the twenty-first century. Many molecular-targeted drugs were investigated and approved for the treatment of malignant diseases, including hematologic diseases and solid tumors. Epidermal growth factor receptor (EGFR) is one of promising molecular targets for treating solid tumors including SCCHN, as the activation of EGFR is observed in SCCHN [18]. With this rationale, many clinical trials of EGFR-targeted therapy for SCCHN were designed and performed.

### Cetuximab – the first molecular targeted drug for head and neck cancer

Cetuximab is an EGFR-targeted monoclonal antibody that improved the OS of SCCHN patients in both locally advanced and recurrent/metastatic settings. The phase 3 IMCL-9815 (the Bonner trial) of locally advanced SCCHN patients compared radiotherapy with or without cetuximab [19]. The median OS was 49.0 months in the patients treated with cetuximab and 29.3 months in those not treated with cetuximab (hazard ratio [HR] 0.74, *p* = 0.03) [19]. The phase 3 EXTREME trial of recurrent/metastatic SCCHN patients compared the efficacy of combination chemotherapy (5-FU + platinum) with cetuximab to that without cetuximab, and the OS was 10.1 months in the patients treated with cetuximab and 7.4 months in those not treated with cetuximab (HR 0.80, *p* = 0.04) [20]. Based on these clinical data, cetuximab was approved in the U.S., the Europe and Japan for the treatment of SCCHN and has been used as the standard treatment option for SCCHN.

There are some unanswered questions regarding the appropriate use of cetuximab in clinical practice, especially for locally advanced SCCHN patients. In the Bonner trial, the superiority of cetuximab + radiotherapy (bioradiotherapy; BRT) compared to radiotherapy was observed, but the result of the trial did not include clinical data comparing BRT with CRT. After the Bonner trial, a randomized phase 2 trial (the TREMPLIN) comparing the efficacy of CRT and BRT for the treatment of SCCHN patients after induction chemotherapy revealed the non-inferiority of BRT [21], and another randomized phase 2/3 trial (the H&N 07 trial) evaluated the efficacy of induction chemotherapy with CRT or BRT using four treatment arms; the results demonstrated the inferiority of BRT, though the trial was not designed to compare the statistical significance of CRT and BRT [22].

Considering the risk of platinum’s cumulative toxicity (e.g., neurotoxicity), BRT after induction chemotherapy could be a reasonable treatment strategy. However, the current randomized phase 3 study (GORTEC 2007–02) failed to observe the superiority of induction chemotherapy followed by BRT compared to CRT [23]. The clinical benefit of the combination of CRT and BRT, i.e., radiotherapy with cisplatin and cetuximab, has also not been established. A phase 3 trial comparing CRT with or without cetuximab (the RTOG 0522 trial) did not show survival benefit by adding cetuximab, and this treatment combination resulted in higher rates of adverse events [24]. Another phase 3 study, the GORTEC 2007–01 trial, showed the improvement of progression-free survival (PFS) provided by BRT with cisplatin compared to BRT [25]. However, that trial’s results did not confirm an improvement in the patients’ OS, and the design of the GORTEC 2007–01 trial does not establish the benefit of adding cetuximab to CRT.

The clinical value of cetuximab is more firmly established in the recurrent/metastatic setting than in the locally advanced setting, but the balance of benefits and adverse events needs much more improvement. Adding cetuximab to cytotoxic chemotherapy involves some specific toxicities such as an acne-like rash, hypomagnesemia, and rarely interstitial lung disease [26–28]. Other combination regimens that are considered for patients who cannot tolerate the above-mentioned EXTREME regimen (5-FU + platinum) or who could be candidates for a replacement of the EXTREME regimen are thus being designed. The combination of taxane and cetuximab has provided some prospective data and showed high response rates [29, 30], and a carboplatin and taxane with cetuximab regimen also seems to be a beneficial combination [31].

The results of the phase 3 trials evaluating the efficacy of cetuximab to SCCHN were summarized in Table 1.

**Table 1:** The results of phase 3 trials evaluating the efficacy of cetuximab to SCCHN

Trial	Patient setting	Treatment arm	OS Hazard ratio (95% CI)	***p***-value	Reference
IMCL-9815 (Bonner)	Locally advanced	BRT vs Radiation	0.74 (0.57–0.97)	0.03	[19]
RTOG 0522	Locally advanced	CRT with or without cetuximab	0.95 (0.74–1.21)	0.32	[24]
GORTEC 2007–01	Locally advanced	BRT with or without cisplatin	0.80 (0.61–1.05)	0.13	[25]
GORTEC 2007–02	Locally advanced	BRT with induction chemotherapy vs CRT	1.12 (0.86–1.46)	0.39	[23]
EXTREME	Recurrent/metastatic	Chemotherapy with or without cetuximab	0.80 (0.64–0.99)	0.04	[20]

*BRT: cetuximab with radiation. CRT: platinum-based chemotherapy with radiation

### EGFR inhibitors other than cetuximab

There are many molecular targeted drugs Other than cetuximab that target the EGFR. Remarkable clinical benefits of the use of small tyrosine kinase inhibitors (TKIs) targeting the EGFR (e.g. gefitinib and erlotinib) have been observed in patients with non-small cell lung cancer (NSCLC) [32], but these TKIs did not show meaningful clinical efficacy against SCCHN [33, 34]. The efficacy of the second-generation TKI afatinib for the treatment of SCCHN was evaluated in several phase 3 trials in different settings, but none of the trials showed clinical benefits, preventing this TKI’s approval for treating SCCHN [35–37]. The efficacy of another monoclonal EGFR antibody for treating SCCHN, i.e., panitumumab, was also evaluated in both locally advanced and recurrent/metastatic settings in clinical trials with designs that were similar to the trials of cetuximab [38, 39]. Those trials failed to observe clinical benefits of panitumumab, similar to the cetuximab results.

### Predictive biomarkers of EGFR-targeted therapy

EGFR-targeted antibodies including cetuximab are thought to bind with the EGFRs on the membrane of the tumor cells and have shown antitumor activity, but clinical analyses indicated that the amplification of the EGFRs in tumor membranes, at least those revealed by immunohistochemistry (IHC), is not a predictive factor of the antibody’s effectiveness. Cetuximab was approved to treat colorectal cancer prior to its approval for head and neck cancer, and EGFR amplification revealed by IHC was originally considered an indication for the use of cetuximab or other EGFR-targeted antibodies. In clinical data however, the amplification of EGFR was not concordant with the clinical efficacy, and subsequent analyses revealed that the KRAS or other RAS mutations, which are located downstream of the EGFR signaling pathway, are more closely related to the efficacy of anti-EGFR antibodies as a negative biomarker [40, 41]. In SCCHN, the nonconcordance of EGFR amplification and the response to anti-EGFR therapy was also observed [42]. Unlike colorectal cancer, RAS mutations are rarely observed in SCCHN [43], but a 2017 report suggested that some KRAS variants in SCCHN might affect the efficacy of cetuximab or other antitumor drugs [44].

As discussed in the Introduction, HPV causes some SCCHNs (oropharyngeal carcinoma in particular). HPV-related SCCHN is known to be more sensitive to cytotoxic chemotherapy and radiation therapy [45], and the efficacy of anti-EGFR therapy is also more prominent in HPV-related SCCHN patients [46, 47]. Whether the increase in the patients’ response to cetuximab treatment in HPV-related SCCHN is more significant than the responses to other cytotoxic drugs is not yet known, and a phase 3 trial (RTOG 1016) comparing CRT to BRT in HPV-related SCCHN did not reveal the non-inferiority of BRT to CRT [48].

## Immune checkpoint inhibitors

One of the most important developments in cancer treatment is the emergence of immune checkpoint inhibitors, which have been incorporated in the standard treatment strategies in many malignant diseases such as lung cancer, gastrointestinal cancers, breast cancer, skin cancers, urological cancers, and more [49]. Immune checkpoint inhibitors target programmed death-1 (PD-1) and cytotoxic T lymphocyte antigen-4 (CTLA-4) on the host T cells or programmed death ligand-1 (PD-L1) on the tumor cells, which brings about the activation of T cells. The immune checkpoint inhibitors have been expected to be effective for treataing cancers with a high tumor mutation burden (TMB), and SCCHN is known to have a high TMB due to its relation to smoking [50]. Considering the clinical benefits of using immune checkpoint inhibitors for SCCHN, many clinical trials have been investigating several immune checkpoint inhibitors for SCCHN.

### Clinical evidence of immune checkpoint inhibitors’ effects in head and neck cancer patients

In a phase 3 trial (CheckMate-141) of SCCHN patients who were refractory to platinum-based chemotherapy, the anti-PD-1 antibody nivolumab provided OS that was superior to physicians’ choices (cetuximab, docetaxel or methotrexate); the OS of the nivolumab arm was 7.5 months, and that of physicians’ choices arms were 5.1 months (HR 0.70, *p* = 0.01) [51]. In a similar setting (the KEYNOTE-040), the anti-PD-1 antibody pembrolizumab also tended to extend the patients’ OS compared to the physicians’ choices, although a significant improvement was not observed [52]. However, an OS benefit was established for pembrolizumab in an untreated recurrent/metastatic setting; in a randomized phase 3 trial (KEYNOTE-048) that compared pembrolizumab monotherapy, a pembrolizumab combination with platinum (cisplatin or carboplatin), and 5-FU to the EXTREME regimen, the pembrolizumab monotherapy, the combination with platinum (cisplatin or carboplatin), and 5-FU provided OS that was superior to that achieved in the EXTREME in PD-L1-positive patients, based on the combined positive score (CPS); the non-inferiority of pembrolizumab monotherapy and its combination were also observed in the total patient population (Table 2) [53]. An anti-PD-L1 antibody that blocks PD-L1 on the surface of tumor cells is also being investigated for SCCHN management, but durvalumab, and anti-PD-L1 antibody which had already been approved to treat lung cancers [54, 55] did not succeed in showing a significant survival benefit for patients with recurrent/metastatic SCCHN in a phase 3 trial [56].

**Table 2:** The overall survival of the patients in the KEYNOTE-048 phase 3 trial comparing monotherapy with the anti-PD-1 antibody pembrolizumab and combination chemotherapy with cetuximab [53]

Treatment arm	Patients	OS (95%CI)	OS of control arm* (95%CI)	HR (95%CI)	***p***-value	Significance	Confirmed result
Pembrolizumab monotherapy	All	11.6 (10.5–13.6)	10.7 (9.3–11.7)	0.85 (0.71–1.03)	0.0456	HR < 1.2	Non-inferiority
	CPS > 1%	12.3 (10.8–14.9)	10.3 (9.0–11.5)	0.78 (0.64–0.96)	0.0086	0.0109	Superiority
	CPS > 20%	14.9 (11.6–21.5)	10.7 (8.8–12.8)	0.61 (0.45–0.83)	0.0007	0.0024	Superiority
Pembrolizumab combination	All	13.0 (10.9–14.7)	10.7 (9.3–11.7)	0.77 (0.63–0.93)	0.0034	0.0041	Superiority
	CPS > 1%	13.6 (10.7–15.5)	10.4 (9.1–11.7)	0.65 (0.53–0.80)	< 0.0001	0.0026	Superiority
	CPS > 20%	14.7 (10.3–19.3)	11.0 (9.2–13.0)	0.60 (0.45–0.82)	0.0004	0.0023	Superiority

*Combination chemotherapy with platinum (cisplatin or carboplatin), 5-FU and cetuximab. CPS: combined positive score

In addition to the recurrent/metastatic settings, the uses of immune checkpoint inhibitors for locally advanced SCCHN are under investigation. The induction of immune checkpoint inhibitors to locally advanced solid tumors is being investigated in many malignant diseases [57], and the safety of immune checkpoint inhibitors was shown in a single-arm clinical trial for the SCCHN oral cavity cancer [58]. It is considered that the combination of radiation therapy with immune checkpoint inhibitor might have adaptive potential, including so-called “abscopal effect” [59], so many clinical trials have been tried to confirm clinical benefit with the combination of radiotherapy and immune checkpoint inhibitors. Clinical trials of immune checkpoint inhibitors combined with radiation therapy were first performed for patients who were intolerable to cisplatin [60], and combinations of immune checkpoint inhibitors with CRT and BRT were subsequently examined [61, 62]. The results of these trials indicated that the use of immune checkpoint inhibitors with CRT would be safe, but the efficacy of such regimens has not been established. The first phase 3 trial (JAVELIN Head & Neck 100), which compared CRT with or without the anti-PD-L1 antibody avelumab, failed to show the superiority of adding avelumab to CRT [63].

The results of phase 3 trials of immune checkpoint inhibitors to SCCHN other than KEYNOTE-048 were summarized in Table 3.

**Table 3:** The results of phase 3 trials evaluating the efficacy of immune checkpoint inhibitors to SCCHN other than KEYNOTE-048

Trial	Patient setting	Treatment arm	OS Hazard ratio (95% CI)	***p***-value	Reference
JAVELIN Head & Neck 100	Locally advanced	CRT with or without Avelumab	1.31 (0.93–1.85)	0.937	[62]
CheckMate 141	Recurrent/metastatic, 2nd line	Nivolumab vs physicians’ choice*	0.70 (0.51–0.96)	0.01	[51]
KEYNOTE-040	Recurrent/metastatic, 2nd line	Pembrolizumab vs physicians’ choice	0.80 (0.65–0.98)	0.0161	[52]
EAGLE	Recurrent/metastatic, 2nd line	Durvalumab vs standard care**	0.88 (0.72–1.08)	0.20	[56]
		Durvalumab plus tremelimumab vs standard care	1.04 (0.85–1.26)	0.76	

*Methotrexate, docetaxel or cetuximab. **Methotrexate, docetaxel, paclitaxel, cetuximab, 5-fluorouracil, TS-1 or capecitabine

### The predictive markers of immune checkpoint inhibitors’ effects

Immune checkpoint inhibitors have improved the outcomes of many cancer patients, but not all patients could benefit from them. Predictive biomarkers of immune checkpoint inhibitors (whether universal or specific to a given disease) have been pursued [64]. The most well-studied biomarker is the PD-L1 expression of tumor cells. PD-L1-positive patients have tended to be more sensitive to immune checkpoint inhibitors in many cancers, but the evaluation methods and/or the threshold for sensitivity have differed among the diseases [65, 66]. PD-L1-positive patients with SCCHN were reported to benefit from immune checkpoint inhibitors much more than those with other types of cancer. Based on the data of the KEYNOTE-048 trial of pembrolizumab, an evaluation of PD-L1 using the CPS criteria is recommended when selecting the treatment regimen, i.e., pembrolizumab monotherapy or a combination [53].

Unlike the scenario concerning cytotoxic drugs and EGFR inhibitors, it remains unknown whether a patient’s HPV infection status affects the efficacy of immune checkpoint inhibitors for SCCHN. A sub-analysis of the CheckMate-141 phase 3 trial of nivolumab suggested that patients with HPV-related SCCHN have a better prognosis when treated with nivolumab [51], but the efficacy of pembrolizumab against HPV-related SCCHN seems no different from that in non HPV-related SCCHN patients [53, 67].

Other biomarkers related to cancer immune are being investigated [68], but an appropriate new marker that can be made available in clinical practice has not been established. From the clinical viewpoint, there is a report describing a difference in patient responses by the tumor lesion site (primary, lymph node metastasis, lung, or other distant metastases) [69].

## Therapy targeting non-squamous cell carcinoma

Most head and neck cancers are SCC as mentioned above, but the other 10% of head and neck cancers has shown various histologies based on the primary organs in the head and neck area. Due to these carcinomas’ rarity and diversity, the clinical evidence of the efficacy and safety of systemic therapies for non-squamous head and neck carcinomas has been insufficient. In clinical practice, head and neck cancers are often treated based on the treatment strategies for SCC. However, there is evidence that supports a specific approach to non-SCC head and neck cancers, and the progress of precision medicine and targeted therapy for these cancers continues.

### Nasopharnygeal carcinoma

Nasopharyngeal carcinoma arises from the nasopharyngeal epithelium, and it is distinguished from other SCCHNs based on it anatomical and etiological factors. These tumors’ origins are deep and adjacent to vital organs such as the eyes, ears and skull base, and thus surgical resection is not indicated. The etiology of nasopharyngeal carcinoma is highly related to EBV infection, and there are diverse differences in its incidence by geographic region: > 50% of nasopharyngeal carcinoma patients are diagnosed in east and southeast Asia [3]. These factors have resulted in nasopharyngeal carcinoma being excluded from many clinical trials of SCCHN, but on the other hand, there is evidence based on randomized clinical trials enrolling only nasopharyngeal carcinoma patients that indicates standard treatment strategies for nasopharyngeal carcinomas.

For locally advanced nasopharyngeal carcinomas, concurrent chemoradiotherapy with cisplatin is the standard, as for SCCHN. However, there are differences in the clinical significance of adjuvant chemotherapy prior to or after CRT; based on the 0099 phase 3 trial comparing the use of CRT after adjuvant chemotherapy with three courses of 5-FU and cisplatin to radiation-only in nasopharyngeal cancer patients [70], the addition of adjuvant chemotherapy remains the standard treatment, although a later randomized trial comparing CRT with adjuvant chemotherapy to CRT-only failed to show significant superiority of adjuvant chemotherapy [71]. Induction chemotherapy prior to CRT for nasopharyngeal cancer was investigated, and two randomized trials in which dose-adjusted TPF (taxane, platinum and 5-FU) and GC (gemcitabine and cisplatin) were evaluated revealed survival benefits of induction chemotherapy for nasopharyngeal carcinoma [72, 73]. Gemcitabine-based chemotherapy also showed efficacy against recurrent/metastatic nasopharyngeal carcinoma [74].

As is the situation for molecular targeted drugs, EGFR-targeted therapy has been anticipated to be effective for nasopharyngeal cancer, and drugs that are specific to nasopharyngeal cancer (e.g., nimotuzumab) are being investigated [75]. Immune checkpoint inhibitors are also speculated to be effective for the treatment of nasopharyngeal cancer, and some small prospective trials have shown promising responses [76–78].

### Salivary gland carcinoma

Salivary gland carcinoma is rare but has heterogenous histological groups [79]. The variety of the histology makes it difficult to obtain evidence regarding the treatment of salivary gland carcinomas in large-scale randomized clinical trials. Molecular targeted drugs approved for the treatment of SCCHN, i.e., EGFR inhibitors and immune checkpoint inhibitors, were also tested for salivary gland carcinoma, but their efficacy was limited to modest responses [80, 81].

In some salivary duct carcinomas, like breast cancers, the expressions of androgen receptor (AR) or human epidermal growth factor 2 (HER2) were observed to be positive, and AR-targeted hormonal therapy and a HER2-targeted antibody showed high response rates [82, 83]. HRAS-targeted therapy for HRAS-mutant cases was recently investigated [84]. Future systemic therapies for salivary gland carcinoma might be designed to be specific to each of this cancer’s histological subtypes.

### Thyroid carcinoma

Thyroid carcinoma has both clinically and pathologically distinct features, and thus the management strategies for this cancer have been distinguished from those for SCCHN, especially for non-surgical treatments. The incidence of thyroid cancer has recently risen, due mainly to the unintended detection of non-clinically relevant nodules by improved imaging technology [85].

The most common histology of thyroid cancers is a differentiated cancer (DTC; differentiated thyroid cancer) including papillary carcinoma and follicular carcinoma, which have indolent prognoses even in the metastatic setting. The need for and the timing of invasive treatment for the management of DTC patients have therefore been discussed in clinical practice [86, 87]. Since DTCs are resistant to the traditional cytotoxic drugs, radioiodine therapy had long been the only systemic therapy for DTC [88]. In the 2010s, several TKIs provided a clinical response and the prolongation of survival, and they were approved in the U.S., the Europe and Japan for practical use [89, 90]. A problem is that the decision regarding when to start treatment with a TKI is not clear, because the disease progression of DTCs can be quite slow. Clinical trials have included DTC patients who were resistant to radioiodine and showed disease progression within the past 12–14 months, and the appropriate follow-up is necessary for DTC patients before introducing a TKI treatment. It was revealed that some DTC patients have BRAF mutation, and BRAF-targeted drugs have been investigated [91].

Medullary thyroid cancer (MTC) is known to be related to multiple neuroendocrine neoplasia type 2 (MEN2), but 75% of the onsets of MTC are sporadic, and MTC causes the secretion of several hormones such as calcitonin, resulting in diarrhea and/or other symptoms [92]. RET mutation has a close relationship with MTC (both hereditary and sporadic), and thus TKIs targeting RET have been investigated. Vandetanib was the first TKI that showed a survival benefit in a phase 3 trial (the ZETA trial) and was approved in the U.S., the Europe and Japan for the treatment of MTC [93]. Cabozantinib, targeting multiple tyrosine kinases RET, also improved the survival of patients with MTC [94], especially those with RET M918T mutation [95]. Treatment with the new RET inhibitor selpercatinib (LOXO-292) was reported to provide a high response rate to MTC [96].

In contrast to DTC, anaplastic thyroid cancer (ATC) is an extremely aggressive cancer, and it is known as one of the poorest-prognosis diseases. Conventionally, various cytotoxic chemotherapies including cisplatin, docetaxel or doxorubicin were tried, but they had limited clinical benefits [97]. TKIs approved to treat DTCs (e.g., sorafenib and lenvatinib) have some clinical data of efficacy against ATC, and the ATC patients achieved modest responses with these agents [98, 99]. As with DTC, there are ATC patients with BRAF mutation, and targeting the BRAF signaling pathway might help improve the survival of ATC patients [100]. Immune checkpoint inhibitors as a treatment for ATC are also being investigated [101].

## Future directions

### Potential new targets in head and neck cancer

Although targeted therapies such as EGFR inhibitors and immune checkpoint inhibitors have been approved as treatments for head and neck cancers, some patients are resistant to these therapies, and thus the searching for new molecular targets continues. Vascular epidermal growth factor (VEGF), an angiogenetic factor on the cell membrane and one of the most well-studied targets for solid tumors [102], was a candidate for the targeted therapy of SCCHN, but the phase 3 trial of the anti-VEGF antibody bevacizumab did not show a survival benefit for SCCHN patients [103]. An assessment of the molecular landscape of SCCHN indicates that the downstreams of molecular growth factors might be candidates for new targeted therapies against SCCHN, such as phosphoinositide-3-kinase (PIK3CA), cyclin-dependent kinase (CDK), or the WNT signaling pathways [104]. There are prospective clinical trials of a PIK3CA inhibitor and a CDK inhibitor as treatments for SCCHN [105, 106].

New immunologic treatments are also under investigation. Regarding combinations with immune checkpoint inhibitors, an indoleamine 2, 3-dioxygenase 1 (IDO1)-targeted therapy was a promising candidate [107], but the negative results of the phase 3 trial in malignant melanoma lessened the motivation for testing this target [108]. Aside from systemic drugs, viral therapy and cell infusion therapy as immune anticancer therapy for head and neck cancer were also investigated [109, 110].

### Local therapy with a molecular targeted strategy

The local control of head and neck cancer directly affects both the survival and the quality of life of the patients, and thus new targeted therapies focusing on the control of the primary site or local recurrence have been examined as head and neck cancer treatments. Boron neutron capture therapy (BNCT), which is radiotherapy by neutron irradiation, targets boron compounds infused and selectively taken into tumor cells [111]. BNCT has been used in clinical practice in Finland [112], and it was approved in Japan in 2020 for the treatment of locally advanced/recurrent head and neck cancer. The published clinical data is limited to small cohorts, but they showed high response rates; one Japanese clinical trial showed 90% overall response rates to 20 head and neck cancers, including 10 locally recurrent SCC, 7 locally recurrent non-SCC and 3 locally advanced non-SCC patients [113]. The availability and feasibility of re-irradiation to locally recurrent lesions which had been already treated with definitive radiation are characteristic in BNCT.

Near-infrared photoimmunotherapy (NIR-PIT) is another new local treatment strategy approved in Japan in 2020, which was developed with the progress of the photothermal and photodynamic therapies for cancers [114]. NIR-PIT consists of infusing a photo-activating chemical and the exposure of the NIR light. The photo-activating chemical includes an antibody that combines tumor cells (EGFR antibody in head and neck cancers) and a photo-absorbing dye, and the absorption of the NIR light induces a ligand-release reaction and even an antitumor immune reaction [115, 116]. In phase 2a trial, NIR-PIT showed 50% of overall response rates to locally recurrent SCCHN patients who could not be satisfactory treated with other local therapies (*n* = 30) [117].

The clinical evidence of these new targeted local therapies is limited to small prospective trials, but in the future some of these therapies might replace conventional radiation therapy or even surgical treatment. For the cure of head and neck cancers and the preservation of patients’ quality of life, both systemic and locally targeted treatments for head and neck cancers should be developed.

## Data Availability

Not applicable.

## Abbreviations

ATC: Anaplastic thyroid cancer
BRT: Bioradiotherapy
BNCT: Boron neutron capture therapy
CDK: Cyclin-dependent kinase
CPS: Combined positive score
CRT: Concurrent chemoradiotherapy
CTLA-4: Cytotoxic T lymphocyte antigen-4
DTC: Differentiated thyroid cancer
EBV: Epstein-Barr virus
EGFR: Epidermal growth factor receptor
5-FU: 5-fluorouracil
GC: Gemcitabine and cisplatin
HER2: Human epidermal growth factor-2
HPV: human papilloma virus
IDO1: Indoleamine 2, 3-dioxygenase 1
IHC: Immunohistochemistry
MEN2: Multiple neuroendocrine neoplasia type 2
MTC: Medullary thyroid cancer
NIR-PIT: Near-infrared photoimmunotherapy
OS: Overall survival
PD-1: Programmed death-1
PD-L1: Programmed death ligand-1
PFS: Progression-free survival
PIK3CA: Phosphoinositide-3-kinase
PS: Performance status
QOL: Quality of life
SCC: Squamous cell carcinoma
SCCHN: Squamous cell carcinoma of the head and neck
TKI: Tyrosine kinase inhibitor
TMB: Tumor mutation burden
TPF: Taxane, platinum and 5-FU
VEGF: Vascular epidermal growth factor
